# 

**Published:** 1857-04

**Authors:** 


### THE SANITARY REVIEW.

### CONTENTS OF No. IX.

Medical Scientific Reform . . . . . . . 1
The Plague at Dunster in 1645: Remarkable Sanitary Provision . . 10

**EPITOME OF SANITARY LITERATURE:**

Treatment of Scarlet Fever . . . . . . 13
Caries of the Teeth . . . . . . *ib.*
Copper Smoke and its Effects . . . . . 14
Meteorological Report . . . . . . *ib.*
Meteorology . . . . . . . 1[illegible]
The Measle of the Pig . . . . . . *ib.*
Qualities of Waters in St. George's, Hanover Square . . . 16
Miscellaneous Works . . . . . . . *ib.*

**ORIGINAL COMMUNICATIONS:**

Disposal of the Fæcal and other Refuse of London on Rational Principles. By P. B. Ayres, M.D. . . . . . . 17
Proposal for the Drainage of London. By T. Hawksley, M.D. . . 28
Report on the Health of the London Postmen in 1856. By Waller Lewis, M.B. . . . . . . . 37
Notes on Certain Vegetable Poisons. By W. Pickells, M.D. (Continued from vol. ii, p. 382) . . . . . . . 44
Diseases of Colliery Operatives, and their Preventible Causes. By W. I. Cox, Esq. (Concluded from vol. ii, p. 378) . . . . 48
Meteorology and Epidemics of the Seventeenth Century. By J. M. Winn, M.D. . . . . . . . . . 55
Graveyard Statistics of Warwick and Leamington. By John Webster, M.D., F.R.S. . . . . . . . 63
On the Drainage of the Metropolis by a Continuous Water-flow. By G. E. Nicholas, Esq. . . . . . . . 65

**SANITARY AND SOCIAL SCIENCE.**

*Reports on Cities, Towns, and Districts.*—Huddersfield . . 73
Edible Seaweeds . . . . . . . 79
Sanitary Improvements in the Manufacture of Chemicals . . 82
Detection of Ergotized Rye . . . . . . 83
Pauperism in England in 1857 . . . . . *ib.*

**PROGRESS OF EPIDEMICS in Thirty-nine Towns and Districts, contributed specially, by the Staff of Epidemic Observers** . . . . 84

**SANITARY LEGISLATION.**

Diseased Animal Food . . . . . . . 104

Medical Organization of the Russian Army . . . *ib.*

The Drainage System . . . . . . . *ib.*

**TRANSACTIONS of the EPIDEMIOLOGICAL SOCIETY of LONDON:**

Climate of the Crimea. By W. R. E. Smart, M.D. . . . 1
Quarterly Report of Proceedings . . . . . 24

**NOTES AND CORRESPONDENCE.**

